# Cerebral venous thrombosis in Latin America: A critical review of risk factors, clinical and radiological characteristics

**DOI:** 10.3389/fneur.2022.1017565

**Published:** 2022-10-28

**Authors:** Gabriel Marinheiro dos Santos Bezerra, Yasmin da Silveira Cavalcante, Paulo Roberto Matos-Neto, Joaquim Francisco Cavalcante-Neto, Keven Ferreira da Ponte, Diana Aguiar de Sousa, Paulo Roberto Lacerda Leal, Espártaco Moraes Lima Ribeiro

**Affiliations:** ^1^Faculty of Medicine of Sobral, Federal University of Ceará, Sobral, Brazil; ^2^Department of Neurosurgery, Federal University of Ceará, Sobral, Brazil; ^3^Stroke Unit, Centro Hospitalar Universitário de Lisboa Central, Lisbon, Portugal; ^4^Department of Neurology, Federal University of Ceará, Sobral, Brazil

**Keywords:** cerebral venous thrombosis, venous sinus thrombosis, stroke, Latin America, South America, Central America, Caribbean, critical review

## Abstract

**Background:**

Cerebral venous thrombosis (CVT) is a rare disease that frequently occurs in young women of childbearing age, with variable clinical presentation in regions with limited access to diagnostic imaging or specialized neurological care. In the last decade, there has been an increase in the number of studies on CVT in Latin America, which may contribute to a better epidemiological description of the disease in this region and, consequently, its early diagnosis.

**Objectives:**

Our study aims to review the risk factors, clinical and radiological characteristics of CVT in Latin America, being critically compared with data from world literature.

**Methods:**

PubMed, ScienceDirect, BVS, and Scopus were searched to identify studies reporting CVT in Latin American countries published up to July 2022. We excluded case reports and case series reporting <5 patients later in the final analysis.

**Results:**

We identified a total of 3714 studies and 26 qualified for the quantitative analysis, which described 1486 cases of CVT. Headache was the most frequent symptom (82.1%) and the use of oral contraceptives in women was the main risk factor (46.7%). The transverse sinus was the most frequent location of the thrombus (52%). The treatment used most in the acute phase was heparin (88.5%) and oral anticoagulation was widely used at hospital discharge (67.8%). The mortality was low (6.5%), and most patients achieved complete recovery (75.3%).

**Conclusion:**

Despite considerable dissimilarities in studies between countries, particularities were identified in the risk factors of CVT in Latin America compared to other regions of the world.

## Introduction

Cerebral venous thrombosis (CVT) is rare when compared to arterial stroke, being more common in young patients (1). The clinical manifestations have a very variable spectrum, with the most frequent symptom being headache (2). The most prevalent risk factors are transient prothrombotic state in women (e.g., pregnancy, puerperium, and oral contraceptive use), hereditary thrombophilia, and malignancy (3). Neuroimaging has provided new, less invasive diagnostic methods such as computed tomography (CT/CT venography) and magnetic resonance imaging (MR/MR venography) (4, 5), though digital subtraction angiography (DSA) is still a widely used method in the diagnosis of patients with cortical or dubious features on MRI. Its treatment is mainly based on anticoagulants.

Most studies on CVT were carried out in developed countries, whose sociodemographic factors differ significantly from those of Latin American countries. Furthermore, most studies on CVT in Latin America have small samples and often do not describe the method responsible for the definitive diagnosis of CVT or the observed parenchymal alterations. These difficulties, coupled with the fact that most Latin American countries present lower middle incomes, varying levels of health care, and many isolated areas with difficult access to tertiary care (6), justify carrying out a literature review to investigate the specificities of CVT in this population. Therefore, this review is essential due to its possibility of revealing mild patterns and initial presentations of the disease that can contribute to the discussion about delays in its diagnosis. The recognition of these specific patterns by healthcare professionals can reduce the impact of CVT and its complications (7).

## Methods

### Literature review

**Figure 1:**
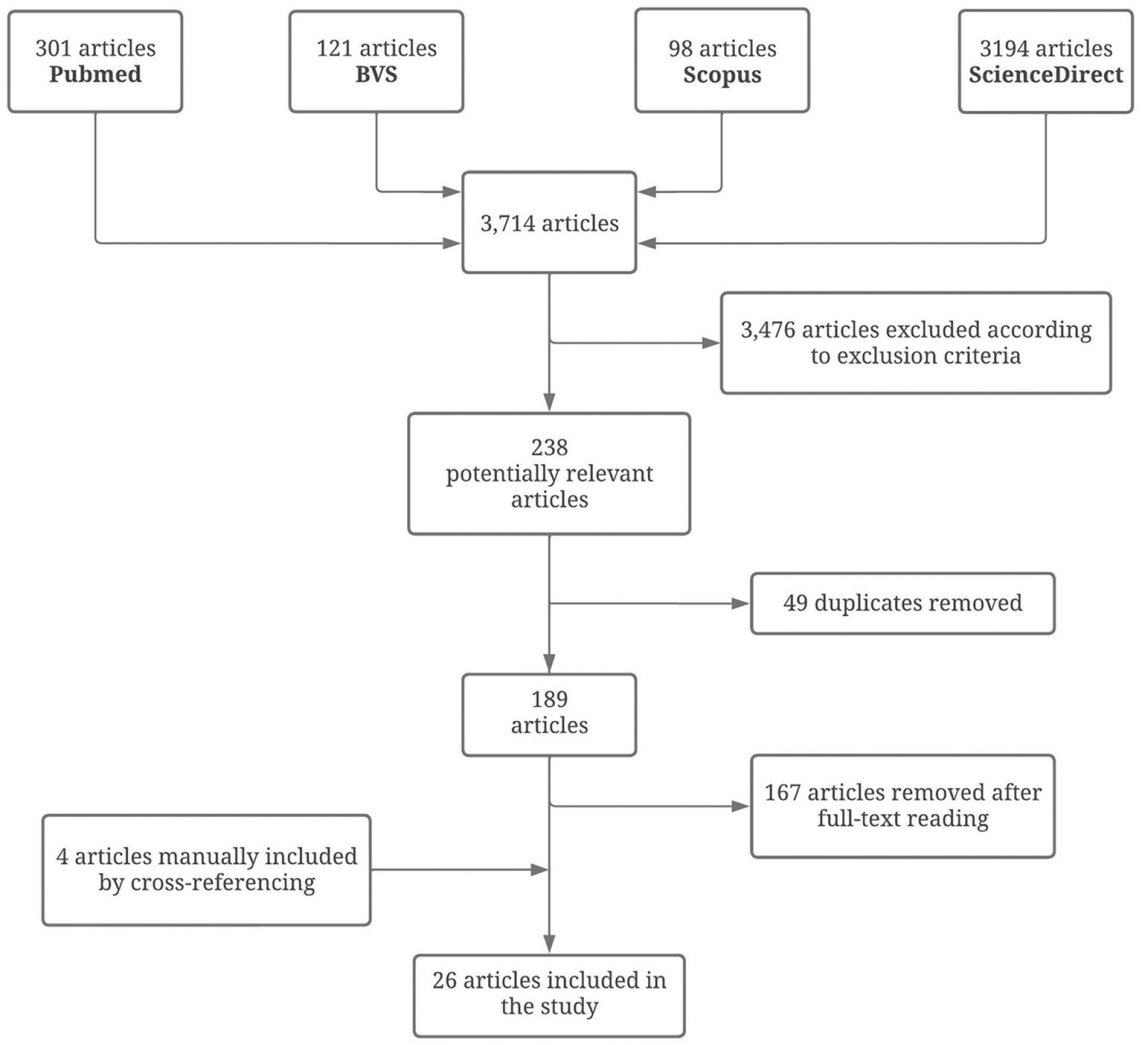
PRISMA flow diagram resuming the literature review process.

We performed a literature review, registered online with PROSPERO (registration number CRD42022331796). PRISMA guidelines were followed for reporting of the review (Figure 1). Three authors (GB, YC, and PR-N) independently extracted data following pre-defined search criteria and quality assessment methods. Disagreements between these authors were resolved by consensus among four authors (GB, YC, PR-N, and JC-N). References obtained by means of research from PubMed, ScienceDirect, BVS, and Scopus were used, with the following search terms: “cerebral venous thrombosis” OR “cerebral vein thrombosis” OR “intracranial sinus thrombosis” OR “thrombosis of the cerebral vein and sinus” OR “cavernous sinus thrombosis” AND searched Latin American country (33 countries, including the Caribbean). The research strategy was designed for MEDLINE and adapted for other databases. Selection was based on the review of the title and abstract of the articles.

Inclusion criteria were: observational studies (case reports, case series, cross-sectional, case-control, and cohort studies) reporting cases of CVT confirmed by imaging diagnostic methods (CT, CT venography, MRI, MR venography, or DSA) in Latin America, without any language restriction, published until July 2022. Samples with <5 patients were later excluded in the final analysis. No limits by sex, age, and ethnicity of study participants or by study type were imposed. Exclusion criteria were: commentaries, editorials, letters, reviews, or articles with only an abstract or title available.

### Data analysis and presentation

The sample group consisted only of patients with a diagnosis of CVT confirmed by imaging methods such as CT, CT venography, MRI, MR venography, or DSA. The percentage of female patients were available in all studies. The following data were described in the studies; age, sex, presentation, risk factors, diagnostic imaging method, imaging findings, treatment, and clinical outcome. If the data described were not available in the studies, they were described as unreported or not available.

## Results

**Figure 2:**
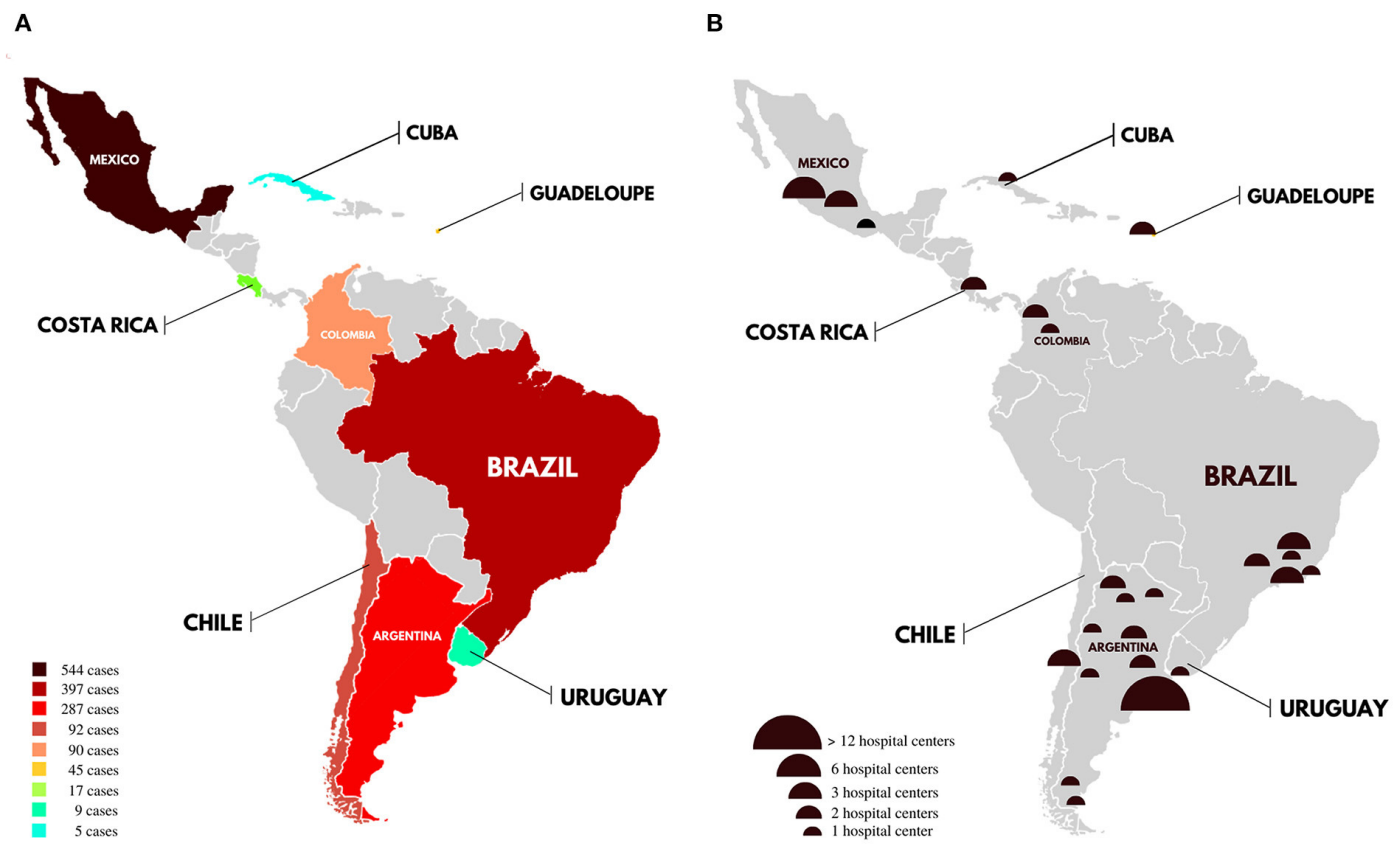
**(A)** Distribution of CVT studies in Latin America. The map shows the reported CVT cases in large series (≥5 patients) by country until July 2022. **(B)** Distribution of CVT database centers in Latin America. The map shows only specified centers in large series (≥5 patients) in each country until July 2022.

The number of patients per study ranged from 5 to 467, with the year of publication ranging from 2003 to 2021. The 26 studies included (8–33) totaled 1,486 patients, with a female predominance (1,157 women, 77.9%), and the mean age was 32.7. The main epidemiological findings in each Latin American country are described in the Supplementary Table. Only 9 Latin American countries were represented (9/36, 27.3%): Argentina (12, 18, 29, 30), Brazil (8, 15, 19, 20, 22, 25, 26), Chile (9, 23, 24), Colombia (13, 31, 33), Costa Rica (28, 32), Cuba (14), Guadeloupe (17), Mexico (11, 16, 21, 27), and Uruguay (10). In Figure 2A, it is possible to identify the reported CVT cases in large series in Latin America. The countries with the most reported patients were Mexico (36.7%), Brazil (26.7%), and Argentina (19.3%). In Figure 2B, the distribution of CVT database centers in Latin America is represented, with emphasis on the Southeast region in Brazil, the Pampeana region in Argentina, and the Central region in Mexico. In addition, studies with great potential of overlapping data (from Argentina, Brazil, and Mexico) were analyzed and selected for a better epidemiological description in this critical review (Supplementary Table). In cases of uncertainty, authors were contacted for further information.

### Clinical presentation

Overall, twenty-one studies (21/26, 80.7%) reported the clinical presentation of patients, totalling 1,164 cases. The most common symptom was headache (887/1081, 82,1%), followed by focal deficits (102/210, 48.6%), nausea/vomiting (87/198, 43.9%), papilledema (273/683, 40%), decreased level of consciousness (including somnolence, stupor, or coma) (331/894, 35.2%), intracranial hypertension (107/370, 28.9%), motor deficits (239/948, 25.2%), cranial nerve disorder (44/181, 24.3%; including cranial nerves II, III, VI, and VII), and seizures (249/1102, 22.6%). The isolated headache rate was 32.5% (95/292). Visual impairment (23/162, 14.2%), aphasia (47/330, 14.2%), and sensory deficits (41/312, 13.1%) were also observed.

### Risk factors

Two studies did not indicate the risk factors associated with CVT. The most commonly reported risk factors were oral contraceptive use (296/634, 46.7% of women) and pregnancy/puerperium (327/991, 33% of women). Thrombophilia was present in 18.1% of the cases (230/1272), the most common presentation having antibodies associated with the antiphospholipid syndrome (59/212, 27.8%), prothrombin G20210A mutation (36/212, 17%), protein S deficiency (31/212, 14.6%), factor V Leiden mutation (25/212, 11.8%), antithrombin III deficiency (21/212, 9.9%), and protein C deficiency (15/212, 7.1%). Other relevant risk factors were obesity (65/309, 21%), family history of thrombosis (11/53, 20.8%), current smoking (88/623, 14.1%) infection (94/723, 13%), and cancer (88/930, 9.5%). Previous thrombotic event (18/198, 9.1%), systemic disease (32/391, 8.2%), trauma (8/161, 5%), and hormonal replacement (11/496, 2.2%) were also reported. Only 48.9% of infections in Latin America had a reported cause, the most frequent being ENT infections (41.3%), including sinusitis and otitis, followed by meningitis (19.6%), sepsis (19.6%) and respiratory diseases (13%). More details are depicted in the Supplementary Table.

### Imaging

MRI/MR venography was the most used method for the diagnosis of CVT in the evaluated studies (275/479, 57.4%), followed by CT/CT venography (141/328, 43%) and DSA (61/322, 18.9%). The CVT location was most commonly reported in the transverse sinus (356/685, 52%), followed by the superior sagittal sinus (400/788, 50.7%), sigmoid sinus (251/812, 30.9%), straight sinus (59/542, 10.9%), cortical veins (28/354, 7.9%), inferior sagittal sinus (11/218, 5%), and cavernous sinus 10/340, 2.9%). The transverse sinus side was described as right in 49.5% of cases (95/192) and left in 55.7% (107/192). The sigmoid sinus side was described as right in 49.5% of cases (55/111) and left in 54% (60/111). The CVT was located in the deep venous system in 10.6% (54/511) of cases and had multiple involvements in 46.5% (290/623) of cases confirmed by any imaging method. The main parenchymal alteration was intracerebral hemorrhage (295/790, 37.3%), followed by venous infarct (325/964, 33.7%) and edema (11/33, 33.3%).

### Treatment

Most articles (20/26, 76.9%) addressed the topic of treatment, with 1,300 patients having their treatments described. Most patients underwent anticoagulation therapy (990/1300, 76.2%). Of these, 3.8% received anticoagulant therapy without specifying the drugs used. Among the articles that detailed the medication in the acute phase, the most used type of heparin was low-molecular-weight heparin (143/365, 39.2%), followed by unfractionated heparin (86/365, 23.6%) and calciparin (13/365, 3.6%). Another 24.4% (89/365) reported having been treated with heparin, but without a description of which type. Oral anticoagulants were used in 67.8% (811/1196) of these patients. Surgical procedures were performed in 5.4% (39/722) (endovascular treatment 1.4%, shunts 1.1%, and hemicraniectomy or craniectomy 0.7%). In addition, other relevant drugs were antiepileptics (16/75, 21.3%), antiplatelet drugs (10/76, 13.2%), antibiotics (7/69, 10.1%), and fibrinolytics (1/34, 3%).

### Outcome

Among the articles that specified when the evaluation was performed (8/26, 32%), the hospital discharge record was the most used (5/8, 62.5%), followed by 30-day (2/8), 90-day (2/8), and 1-year (2/8) follow-up. Only 3 studies recorded the outcome in more than one moment. Most patients had their recovery assessed by the modified Rankin scale (mRS) (894/1012, 88.3%), with 0-2 being the most frequent score to assess complete recovery. In addition to the use of mRS, another evaluation method of evolution distinguishing between those who had or did not have neurological sequelae and were free of disease symptoms or not was used in 8.3% of patients (84/1012). The Pediatric Stroke Outcome Measure (PSOM) to assess the result (34/1012). Only 3 articles described CVT recurrence, in which 11 patients (11/410, 2.7%) had CVT recurrence. 6.5% (73/1121) of patients with reported outcomes died. Among patients with detailed results, 7.5% had partial recovery (76/1017), and 75.3% had complete recovery (766/1017). Only 2 patients were lost to follow-up (2/1121, 0.2%).

## Discussion

In this critical review of 26 studies and nearly 1,500 patients, the risk factors and clinical-radiological characteristics of CVT in Latin America were described. The proportion of women affected in our data (77%) was higher compared to large studies in world literature (rate 59–74.5%) (1, 3, 4, 34). Previously, only one review addressed this issue, but it was restricted to 79 patients and to South America. The possibility of carrying out new studies in each country, for example prospective cohort studies, could improve the discussion on the description of CVT in Latin America. The main findings from the population analysis were as follows: (1) pregnancy/puerperium was a significant risk factor for CVT; (2) papilledema was a very frequent clinical manifestation in large CVT studies in Latin America, which may represent a delay in the diagnosis of patients; (3) there are great opportunities for treatment and improving the diagnosis of CVT in Latin America, with emphasis on increased rates of treatment with anticoagulation and of use of MRI as a diagnostic method. Furthermore, our data suggests that the identification of acquired or genetic thrombophilia may have been underestimated in many studies, which may be related to the lack of adequate tests.

### Clinical presentation

About 80% of patients presented headache, which demonstrates the importance of this symptom for the construction of diagnostic reasoning. In sequence, focal deficits and nausea/vomiting were reported. Few studies reported isolated headache (23%), however it was an important symptom associated with CVT when reported.

Papilledema is an important sign of intracranial hypertension, being present in 40% of patients, an even higher rate in relation to the International Study on Cerebral Vein and Dural Sinus Thrombosis (ISCVT) (1). This demonstrates not only the importance of the ophthalmological examination in CVT, due to its low cost and capacity to identify this important clinical sign of intracranial hypertension, but more importantly a possible delay in the diagnosis of these patients, as the appearance of papilledema usually occurs after other manifestations of CVT (7). It is worth mentioning that the studies did not detail the time until the diagnosis of CVT.

### Risk factors

The most common risk factor in our study was the use of oral contraceptives in women. The use of drugs with a lower risk of thrombogenicity could reduce the CVT rates in these cases. Furthermore, in this review pregnancy/puerperium had a higher rate (33%) than in continental multicenter studies (range 8–19%) (1, 4, 34). Mexico seems to contribute considerably to this disparity. Regional differences related to access to maternal and childbirth health services, including the detection of other risk factors, such as thrombophilia, may contribute to this heterogeneity of data. Only one Brazilian study analyzed the ethnic differences associated with CVT in Latin America, suggesting a severe disease at onset greater in blacks and mullatoes than whites (19). These findings are similar to those found in an arterial stroke study (35).

Regarding acquired or congenital thrombophilia, 27.8% of patients with defined thrombophilia had antibodies related to antiphospholipid syndrome. Furthermore, the prothrombin G20210A mutation and protein S deficiency were the most commonly detected hereditary thrombophilia in this study. In Europe population, a previous study of hereditary thrombophilia showed a different pattern, with greater rates of factor V Leiden mutation (36). At this time, data are not sufficient to affirm that these differences are of ethnic origin, but ethnic variations most probably contribute. Among those studies in which patients were tested for thrombophilia, few reported whether the entire sample was tested or whether there was overlap between thrombophilia types (10, 15, 18, 21, 22). In low- and middle-income regions, ENT infections seem to have high prevalence rates (7), a fact also observed in this study. Thus, socioeconomic factors may be involved. However, the rate of infections in this study (13%) was only slightly higher than in global studies (range 8–12.3%) (1, 3, 4, 34).

### Imaging

MRI was the most used diagnostic method in our study. This is possibly a reflection of the advances in neuroimaging technology, which has led to the widespread use of MRI in middle- and even low-income countries, facilitating the diagnosis of CVT and improving the overall prognosis of patients (5). However, the rate of MRI use in Latin America (57.4%) was lower than rates in relevant international studies (range 71-89.1%) (1, 3, 34), still demonstrating the need for greater access to this imaging technique in Latin America. In Mexico, the study with the largest sample did not clarify in the data on the imaging method, impairing a more detailed analysis in this country.

The location of the thrombosis was described mostly in the transverse sinus. Large studies around the world already demonstrate this pattern of involvement with higher rates (range 72–86%) (1, 3, 4). On the other hand, important studies report greater involvement of the superior sagittal sinus (34, 37). Thus, the cause of this variability is not clarified in the literature. Intracerebral hemorrhage was the main parenchymal alteration observed in our study (37.3%), with a similar prevalence to European data (36%) (1). In addition, few articles (23.1%) specified the type of venous infarction (hemorrhagic or non-hemorrhagic).

### Treatment

The therapeutic modality was one of the most consistent pieces of information in the evaluated studies and one of the strengths of this review. Thus, many articles indicated that the most used treatment in the acute phase was heparin, especially low-molecular-weight heparin, although unfractionated heparin was also widely used and some studies did not detail the type of heparin administered (13, 15, 20, 31). Therefore, most patients had access to an effective and low-cost medication, despite the limited economic conditions of some Latin American regions. At hospital discharge, oral anticoagulants (mainly vitamin K antagonists) were prescribed to most patients (67.8%) for a period ranging from 3 to 12 months. This rate is lower than in large multicenter studies such as ISCVT (83.3%) and CEVETIS (88.8%) (1, 4). This may reinforce the need for an increase in anticoagulation rates in the context of CVT in Latin America.

### Outcome

The most used criterion to describe the evolution of patients was the mRS scale, with an index of 0-2 indicating complete recovery. However, some studies only separated patients into those who remained with neurological sequelae or not, and another used the PSOM scale, suitable for classifying pediatric patients. We chose to respect the criteria adopted by each author and, according to them, we classified patients as those who had complete recovery, partial recovery, or who died, considering the descriptions provided by the authors about recovery and death, without deducing that the remaining patients would have had partial recovery.

In addition, 30% of the studies did not detail the follow-up of the patients, and there was also no unanimity regarding the intervals of this follow-up, as some reported it at 3 months, 6 months, 9 months, or 1 year after hospital discharge. The criterion chosen by each author were used and, in cases of registration in more than one moment, were selected the one with the highest number of patients described. Most patients achieved complete recovery, in more than 75% of cases, even though this percentage is slightly lower than that obtained in large studies in the literature (range 79–88.5) (1, 3, 34). The remaining patients had partial recovery and a minimal amount, just over 6%, died. The high mortality rate of Cuba was considerably atypical and can be related to the small sample size. It is possible to reaffirm the effectiveness of the treatment and the low mortality from CVT, even in countries with lower socioeconomic conditions.

### Limitations

Many articles, even with a previous reading of the title and abstract, did not fit the adopted inclusion criteria, as they did not provide the available article in full for reading or had insufficient data. Another limiting aspect was the possibility of selection biases, as the unavailability of images may have limited access to diagnosis among the poorest. In addition, even with the exclusion of studies with great potential of overlapping data that had database centers in common, the inclusion of studies carried out over a wide period of time and with large samples may have contributed to an overlap of data in the same country. Futhermore, an important limitation is that there were no age and sex restrictions on study participants in our selection of articles. In this context, two articles in our sample (23, 32) included only pediatric patients, while one article (21) only addressed puerperal women, although this did not significantly affect our epidemiological representation. In this review, the unavailability of data (such as mean age, detailed risk factors, or imaging features) was another limiting aspect. In addition, our review included mainly populations from high-middle-income countries (Argentina, Brazil, and Mexico), which does not necessarily translate into the total Latin American population.

## Conclusions

CVT is a rare disease that presents epidemiological particularities in Latin America in relation to other regions of the world. Some specificities were found in this study, such as the significant presence of pregnancy/puerperium as a risk factor for the occurrence of CVT. More knowledge is needed about the association of these rates with the high birth rates existing in underdeveloped regions or with the underreporting of other risk factors in these regions. Another question is that a possible delay in the diagnosis of CVT may exist in Latin American countries, demonstrated by the high rates of papilledema. This review may stimulate the awareness of clinicians working in Latin American countries for more comprehensive studies on CVT.

## Author contributions

GB, YC, PM-N, JC-N, DAS, and ER: conception and design of the work. GB, YC, PM-N, and JC-N: literature search, acquisition, analysis, and interpretation of data for the work. GB, YC, PM-N, JC-N, KP, DAS, PRLL, and ER: drafting the work. All authors were involved in critical revision of the manuscript for relevant intellectual content.

## Conflict of interest

The authors declare that the research was conducted in the absence of any commercial or financial relationships that could be construed as a potential conflict of interest.

## Publisher's note

All claims expressed in this article are solely those of the authors and do not necessarily represent those of their affiliated organizations, or those of the publisher, the editors and the reviewers. Any product that may be evaluated in this article, or claim that may be made by its manufacturer, is not guaranteed or endorsed by the publisher.
