# Corrigendum: Cerebral venous thrombosis in Latin America: A critical review of risk factors, clinical and radiological characteristics

**DOI:** 10.3389/fneur.2022.1130071

**Published:** 2023-01-05

**Authors:** Gabriel Marinheiro dos Santos Bezerra, Yasmin da Silveira Cavalcante, Paulo Roberto Matos-Neto, Joaquim Francisco Cavalcante-Neto, Keven Ferreira da Ponte, Diana Aguiar de Sousa, Paulo Roberto Lacerda Leal, Espártaco Moraes Lima Ribeiro

**Affiliations:** ^1^Faculty of Medicine of Sobral, Federal University of Ceará, Sobral, Brazil; ^2^Department of Neurosurgery, Federal University of Ceará, Sobral, Brazil; ^3^Stroke Unit, Centro Hospitalar Universitário de Lisboa Central, Lisbon, Portugal; ^4^Department of Neurology, Federal University of Ceará, Sobral, Brazil

**Keywords:** cerebral venous thrombosis, venous sinus thrombosis, stroke, Latin America, South America, Central America, Caribbean, critical review

In the published article, an author name was incorrectly written as “Gabriel Marinheiro dos Santos-Bezerra.” The correct spelling is “Gabriel Marinheiro dos Santos Bezerra.”

In the published article, there was an error. The author initials used in the **Author contributions** section and **Methods** section were incorrect. GS-B should be GB.

A correction has been made to **Methods**, “*Literature review*,” Paragraph 1. The corrected sentence is:

We performed a literature review, registered online with PROSPERO (registration number CRD42022331796). PRISMA guidelines were followed for reporting of the review (Figure 1). Three authors (GB, YC, and PR-N) independently extracted data following pre-defined search criteria and quality assessment methods. Disagreements between these authors were resolved by consensus among four authors (GB, YC, PR-N, and JC-N).

A correction has been made to the **Author contributions**. The corrected statement appears below.

GB, YC, PM-N, JC-N, DAS, and ER: conception and design of the work. GB, YC, PM-N, and JC-N: literature search, acquisition, analysis, and interpretation of data for the work. GB, YC, PM-N, JC-N, KP, DAS, PRLL, and ER: drafting the work. All authors were involved in critical revision of the manuscript for relevant intellectual content.

The authors apologize for this error and state that this does not change the scientific conclusions of the article in any way. The original article has been updated.

